# Exosomes are released by bystander cells exposed to radiation-induced biophoton signals: Reconciling the mechanisms mediating the bystander effect

**DOI:** 10.1371/journal.pone.0173685

**Published:** 2017-03-09

**Authors:** Michelle Le, Cristian Fernandez-Palomo, Fiona E. McNeill, Colin B. Seymour, Andrew J. Rainbow, Carmel E. Mothersill

**Affiliations:** 1 Radiation Sciences Graduate Program, McMaster University, Hamilton, Ontario, Canada; 2 Institute of Anatomy, University of Bern, Bern, Switzerland; 3 Department of Physics & Astronomy, McMaster University, Hamilton, Ontario, Canada; 4 Department of Biology, McMaster University, Hamilton, Ontario, Canada; Northwestern University Feinberg School of Medicine, UNITED STATES

## Abstract

**Objective:**

The objective of our study was to explore a possible molecular mechanism by which ultraviolet (UV) biophotons could elicit bystander responses in reporter cells and resolve the problem of seemingly mutually exclusive mechanisms of a physical UV signal & a soluble factor-mediated bystander signal.

**Methods:**

The human colon carcinoma cell line, HCT116 p53 +/+, was directly irradiated with 0.5 Gy tritium beta particles to induce ultraviolet biophoton emission. Bystander cells were not directly irradiated but were exposed to the emitted UV biophotons. Medium was subsequently harvested from UV-exposed bystander cells. The exosomes extracted from this medium were incubated with reporter cell populations. These reporter cells were then assayed for clonogenic survival and mitochondrial membrane potential with and without prior treatment of the exosomes with RNase.

**Results:**

Clonogenic cell survival was significantly reduced in reporter cells incubated with exosomes extracted from cells exposed to secondarily-emitted UV. These exosomes also induced significant mitochondrial membrane depolarization in receiving reporter cells. Conversely, exosomes extracted from non-UV-exposed cells did not produce bystander effects in reporter cells. The treatment of exosomes with RNase prior to their incubation with reporter cells effectively abolished bystander effects in reporter cells and this suggests a role for RNA in mediating the bystander response elicited by UV biophotons and their produced exosomes.

**Conclusion:**

This study supports a role for exosomes released from UV biophoton-exposed bystander cells in eliciting bystander responses and also indicates a reconciliation between the UV-mediated bystander effect and the bystander effect which has been suggested in the literature to be mediated by soluble factors.

## Introduction

Cells subjected to both non-ionizing and ionizing radiation have the capacity to generate communication signals and subsequently cause biological changes in distant non-irradiated cells [1–5]. This observed phenomenon whereby intercellular communication and biological change is initiated as a result of irradiation is referred to as the *radiation-induced bystander effect* (RIBE). The RIBE has been shown to elicit a spectrum of effects in bystander cells that reflect biological responses which are closely representative of those characterized by directly-irradiated cells. Sister chromatid exchanges, micronuclei formation, apoptosis, genomic instability, and mitochondrial dysfunction have all been demonstrated in bystander cells subsequent to the receipt of signals by directly-irradiated cell populations [6–8].

The communication of bystander signals between directly-irradiated and bystander cells can be accomplished via various mechanisms including the facilitation of molecular exchange between adjacent cells via gap junctions [3], the communication between distant cells via the transfer of soluble factors [2], the exchange of volatile components between physically separated cell populations [9, 10], and the transmission of electromagnetic signals from irradiated cells to distant recipient cells [11–13]. In the study of bystander effects signalled via the exchange of soluble factors, a role has been identified for a variety of signalling molecules such as reactive oxygen species [14], cytokines [15, 16], and exosomes [17] in the generation of bystander responses. The propagation of this bystander mechanism requires either direct physical contact between cells, the exchange of biological fluids, such as blood serum or cell culture media, between the directly-irradiated cells and the non-irradiated bystander cells, or an open system so as to facilitate the exchange of volatile components between two separate organisms or cell populations. In an alternative bystander mechanism, the role of electromagnetic radiation in the ultraviolet (UV) wavelength range has been identified [11–13]. This novel bystander mechanism has been referred to as the *UV-mediated bystander effect* whereby the communication of signals via light fields does not require physical contact between directly-irradiated and bystander cell populations [13].

Cellular communication mediated by electromagnetic radiation occurs as a result of *biophoton* emission by one population of cells and the receipt of those signals by another cell population. Biophotons are characterized by UV and visible wavelength range photons which are emitted from biological materials via processes alternative to conventional chemiluminescence [18]. While the mechanisms for biophoton emission are still unclear, the excitation of various intracellular molecules is a strong candidate mechanism [19, 20]. The initiation of biophoton emission by biological systems has been observed subsequent to stress induction by ionizing radiation [21–24], viral infection [11], and mechanical disruption [25]. While the observed rates of biophoton emission are typically quite low (0.01 photons per second per cell; 100 photons measured per 10^4^ plated cells [26], 10^4^ photons detected per 10^6^ plated cells [13, 23]), and thus the dose delivered to cells may not be considered significant enough to induce visible effects, there is evidence to suggest that biophotons act as coherent information-encoding signals, similar to binary-encoded data, to exchange information between biological systems [18, 19].

The bystander system which the current study has employed to investigate the UV-mediated bystander effect is characterized by the incubation of two separate cell populations in UV-transmitting vessels in order to achieve successful biophoton signal transduction [13]. Briefly, cells in one culture were directly irradiated with beta-emitter, tritium, to induce UV biophoton emission. The biophotons emitted from the tritium-irradiated cells were measured using a photomultiplier tube fitted with interference-type band pass filters and were found to exhibit emission in each of the UV-A (340 ± 5), UV-B (300 ± 5) and UV-C (280 ± 5) wavelength ranges. UV-A photon rates reached 1200 counts per second per 10^5^ cells whereas the UV-B and UV-C wavelengths exhibited weaker photon emission rates following the same given activity of beta radiation [13]. A bystander cell culture was incubated 1.5 cm superior to the directly-irradiated cell monolayer for 24 hours to accommodate biophoton signal receipt. Upon analyzing the clonongenic survival data of the bystander cells which received the UV biophoton signals, it was found, using a Pearson’s correlation test, that 95% of the cell killing observed in the bystander cell population shared a relationship with the measured UV-A biophoton flux. The role of the detected UV biophotons in eliciting the observed bystander responses was further confirmed when the placement of a polyethylene terephthalate UV-absorbing filter between the directly-irradiated and the bystander cell populations effectively abolished cell killing in the bystander population [13]. The UV-mediated bystander effect has since been investigated in the human keratinocyte cell line, HaCaT [13], and human colon carcinoma cell lines, SW48, HT29, HCT116 p53 +/+, and HCT116 p53 -/- [27]. The work of Kaznacheev and colleagues also supports the idea of intercellular communication by electromagnetic means as they demonstrated the ability of virally-infected cell populations to elicit stress responses in non-infected populations only when the two populations were separated by UV-transmitting materials [11]. Although they did not describe this observation using the term “bystander”, the communication between stress-induced cells and nearby reporter cells certainly fits within what we now call the bystander effect. Despite the demonstrated involvement of electromagnetic radiation in the generation of bystander effects in response to various stressors [11–13], the molecular aspects by which the UV-bystander signal exerts its effects upon bystander cells remains unclear and requires further investigation.

This study thus sets out to investigate a molecular mechanism by which UV bystander signals may potentially elicit biological effects in bystander cells. Recent evidence has brought to light the ability of ultraviolet radiation to modulate the function of exosomes emitted from human keratinocyte cells [28]. Cicero *et al*. showed that the exosomes extracted from UV-B-irradiated keratinocyte cells were able to induce greater melanin production by melanocyte cells. While the study by Cicero investigated UV-B radiation, the interactions expected from UV-A photons, as investigated in the current study, are similar to those observed subsequent to UV-B exposure due to their similarity in wavelength and photon energy. Knowledge of UV’s modulatory effect upon exosome function is promising as it may provide a point of reconciliation between the UV-mediated bystander effect and the previously discussed soluble factor-mediated bystander effect. To elaborate, exosomes are extracellular vesicles derived from pinched off sections of the endosomal membrane [29]. These 50–150 nm membrane-bound vesicles [30] encapsulate cytoplasmic contents such as RNA and protein during formation and are subsequently released into the extracellular space. The contents of exosomes can exert their effects upon bystander cells as a result of exosome migration through the extracellular space to distant cells and subsequent internalization of those exosomes by endocytosis. Their ability to efficiently transport essential biological molecules from cell to cell through the intercellular environment emphasizes their significant contribution to soluble-factor-mediated intercellular signalling. Published literature has demonstrated exosomes’ ability to induce carcinogenic behaviour (tumour cell promotion and migration in cells receiving exosomes from gastric tumour cells [31]) and to induce DNA damage in bystander cells receiving exosome-encapsulated RNA from x-irradiated breast cancer cells [17].

The evident role of exosomes in intercellular signalling therefore justifies the consideration of their role in inducing the bystander response. While studies demonstrating the involvement of exosomes in the RIBE already do exist in the published literature [17, 29, 32–34], the investigation of exosomes as they pertain to secondary UV biophotons, is a previously unexplored and novel concept. The rationale for investigating biophotons in relation to exosomes is based upon the hypothesis that UV biophotons may act to elicit the release of a variety of soluble factors that are commonly involved in the RIBE. While there are many soluble factor candidates that could have been selected for investigation, particular focus upon exosomes was chosen because protocols for clean exosome isolation from culture have been well established in the literature. Furthermore, the vesicular nature of exosomes facilitates opportunities for further comprehensive investigation extending beyond the investigations undertaken in the current study. This research aims to assess the potential relationship between UV biophotons and the release of soluble factors in the study of bystander signalling.

The current study investigates the relationship between cellular UV biophoton exposure and the release of exosomes in response to that exposure. The system for investigating bystander effects used in our previous research [13] suggests that soluble factors, including exosomes, cannot be the only signal from directly-irradiated cells driving the bystander effect. Our system did not facilitate any medium transfer or cell-cell contact between the directly-irradiated and bystander populations, yet significant bystander effects could still be observed. With this in mind, we can concede two solutions; either there are two mutually exclusive mechanisms by which the bystander effect can be induced, or the UV signal is able to trigger the release of soluble factors from bystander cells. We hypothesized that exposure of cells to UV biophotons will trigger the release of soluble factors that are subsequently capable of eliciting bystander responses. That is, we believe that the UV biophotons emitted from cells as a result of direct beta-particle irradiation, is an intermediate signal that is responsible for triggering the release of bystander-eliciting soluble factors. The current study has confirmed this relationship via the assessment of clonogenic survival and mitochondrial membrane potential in bystander cells receiving exosomes extracted from UV-exposed cells.

## Materials and methods

### Cell culture

HCT116 p53 +/+ human colon carcinoma cells, received as a gift from Dr Robert Bristow (University of Toronto) and Dr Bert Vogelstein [35], were cultured in RPMI1640 supplemented to a final concentration of 10.5% fetal bovine serum (FBS) (Gibco), 2 mM L-glutamine, 100 U/mL penicillin, and 100 *μ*g/mL streptomycin sulphate. Cells were routinely cultured in 75 cm^2^ flasks (BD Falcon), given medium exchanges every 2 to 3 days, and passaged with 3 mL of 0.25% trypsin-EDTA solution when cells reached 80–90% confluence. Neutralization of the trypsinization process was accomplished by adding 7 mL of complete growth medium to the trypsinized cell suspension. Cell cultures were routinely incubated at 37°C, 95% humidity and 5% CO_2_. All reagents used were from Gibco unless otherwise stated.

Cells were given full volume medium renewals 24 hours prior to an experiment using RPMI1640 supplemented with exosome-depleted FBS (Gibco cat no. A2720801) in place of the FBS used for routine subcultivation. For all cell culture activities carried out through the experimental process, the exosome-depleted growth medium was used. For cells intended to receive direct irradiation and for cells destined to receive ultraviolet (UV) photon signals from beta(*β*)-irradiated cells, 25 cm^2^ flasks containing a total volume of 5 mL complete growth medium were seeded with 2 × 10^5^ cells each. For reporter cells destined to receive either cell-conditioned medium harvested from UV biophoton-exposed cells (UV-ICCM), control cell-conditioned medium (CCCM) or exosomes isolated from either UV-ICCM or CCCM, cells were seeded into 25 cm^2^ flasks at clonogenic densities (500 cells per flask, 5 mL total volume). Use of the term *UV-ICCM* throughout the text refers to cell culture medium that has been conditioned by cells that have been exposed to the *UV biophotons* emitted by *β*-irradiated cells; it does not refer to the cells which have been directly exposed to *β*-particles.

### Direct beta-irradiation and bystander protocol

Beta (*β*)-irradiation of cell cultures containing 2 × 10^5^ cells was accomplished by adding tritiated water directly into the cell culture medium. 857.5 *μ*Ci of pure *β*-emitter, tritium (^3^H), was added into cell culture medium and retained in the medium for 24 hours to achieve a total dose of 0.5 Gy. The (^3^H) dose was determined using Eq 1 where D represents the dose in Joules/kilogram (Gy), N_0_*λ*_R_ is the ^3^H activity in disintegrations per second (Becquerel), ${\bar{E}}_{\beta }$ is the average tritium beta particle energy, t is the duration of the irradiation in seconds, and m represents the mass of the irradiated object. During the 24 hour irradiation period (at 37°C, 95% humidity, 5% CO_2_), 25 cm^2^ bystander flasks each containing 2 × 10^5^ cells were placed directly superior to the petri dishes containing the directly-irradiated cultures such that the bystander cells were in the field of the ultraviolet (UV) photon emissions generated by the directly-irradiated cells but were not directly irradiated by the beta particles from the tritium. The monolayer of directly-irradiated cells was separated from the bystander cell monolayer by a distance of approximately 1.5 cm. The two cultures were incubated together in a partitioned light-tight box to eliminate potential effects from ambient light during the opening of the incubator door and from cross-interference of UV biophoton signals from other directly-irradiated cultures within the light tight box. Controls for the *β*-irradiation trials included bystander flasks placed superior to non-*β*-irradiated (sham) cells and irradiated cell culture medium (without cells).

Immediately following 24 hour irradiation, UV-ICCM and CCCM from bystander flasks were harvested, filtered (0.2 *μ*m pore filter, Pall Corporation), and either transferred to flasks containing clonogenic reporter cells or transferred to polycarbonate ultracentrifuge tubes for exosome extraction.

$$D=\frac{N_{0}\lambda_{R}{\bar{E}}_{\beta }t}{m}$$ (1)

### Exosome isolation

Exosomes were isolated via ultracentrifugation of UV-ICCM or CCCM at 100,000 xg for 90 minutes using a Thermo Scientific WX90 Sorvall Ultracentrifuge with a F50L-8x39mL fixed angle rotor. For the exosome experiments, an additional control was added whereby exosomes were extracted from complete growth medium which was not irradiated nor conditioned by cells to ensure that any observed effects were not attributed to the culture medium itself. The samples were kept at 4°C for the duration of the ultracentrifugation process. Following ultracentrifugation, the supernatant was aspirated and the exosome pellet was resuspended in 250 *μ*L Dulbecco’s Phosphate Buffered Saline (DPBS). Exosome isolates were transported from the lab housing the ultracentrifuge to our cell culture lab on ice and immediately added into the cell culture medium of the reporter cells. These reporter cells were cultured in 5 mL of growth medium supplemented with exosome-depleted fetal bovine serum. The elapsed time from exosome resuspension to addition of exosomes to reporter cells was approximately 20 minutes. The reporter cells were plated at clonogenic densities to assess for survival or plated onto 96-well plates to assess for mitochondrial membrane potential using the JC-1 assay. Remaining exosome fractions were stored at -20°C for future validation of exosome-enriched proteins using the western blot assay.

### Clonogenic survival in reporter cells

The clonogenic survival assay was used to assess the survival of bystander cells which received UV-ICCM, CCCM, or exosomes extracted from UV-ICCM and CCCM. For reporter cells which directly received UV-ICCM or CCCM, the medium that was originally used to culture the reporter cells was discarded and replaced by the full volume of UV-ICCM or CCCM that was harvested. For reporter cells receiving exosome fractions, the cell culture medium originally used to culture the cells was retained and 250 *μ*L of exosome fraction (exosomes extracted from the UV-ICCM of 2 × 10^5^ cells) was added to the existing medium. An additional experiment was also conducted whereby UV-ICCM and CCCM was ultracentrifuged and the supernatant (free of exosomes) was harvested and subsequently placed onto reporter cells.

In the permutation whereby the role of RNA-carrying exosomes was being assessed, RNase was added to and incubated at 37°C for 30 minutes with the volume of UV-ICCM, CCCM or exosome fraction prior to their transfer into the reporter cell culture.

UV-ICCM, CCCM, or exosome fractions were incubated with the reporter cells for approximately 9 days at 37°C, 95% humidity, and 5% CO_2_ to facilitate the growth of single cells into colonies. Reporter flasks were then stained and the quantity of cells which developed into colonies (>50 cells) were scored. The number of colonies formed in the treatment and control flasks were normalized to six plating efficiency flasks for each trial. The plating efficiency flasks were seeded with 500 cells per flask where three were plated at the beginning of the seeding process and three were plated at the end. The average plating efficiency among all three trials (18 plating efficiency flasks) was 33.5% ± 2.5% (standard error of the mean).

### Mitochondrial membrane potential in reporter cells

Mitochondrial membrane potential was assessed in this study to determine the role of exosomes generated as a result of cellular exposure to UV bystander signals in the initiation of apoptosis in reporter cells exposed to those exosomes. For each experimental sample, a 1 mL suspension of 2 × 10^6^ HCT116 p53 +/+ cells was first incubated with 250 μL exosome fraction, 1 μL carbonyl cyanide 3-chlorophenylhydrazone (CCCP) (membrane depolarization positive control), or 1 μL dimethyl sulfoxide (DMSO) (membrane depolarization negative control) for 1 hour at 37°C. Following incubation with treatment samples and subsequent elimination of treatment samples from the cell suspension by 5-minute centrifugation at 1000 rpm and resuspension in complete growth medium, 3.83 μM MitoPT JC-1 reagent from the MitoPT JC-1 mitochondrial permeability assay kit (ImmunoChemistry Technologies, cat no. 924) was incubated with the cell suspension for 15 minutes at 37°C. Following incubation, the cells were washed with DPBS and subsequently pelleted at 1000 rpm for 5 minutes to remove the supernatant containing residual JC-1 stain. The cell pellet was resuspended in 1 mL DPBS and 100 *μ*L of the suspension was subsequently pipetted into the well of a black glass-bottom 96-well plate (BD Falcon) to achieve a total of 2 × 10^5^ cells in each well. Each treatment sample was pipetted into 6 wells such that there were 6 replicates of each sample on a given 96-well plate.

Fluorescence spectroscopy was accomplished using a Tecan Infinite M200 Pro plate reader and i-control software where excitation was set to 488 nm and emission (measurement) wavelengths were set to 590 nm (red) and 527 nm (green). Mitochondrial membrane potential was assessed by taking the ratio of red to green fluorescence indicative of the relative ratio of aggregates to monomers in the cell culture. The concentration of JC-1 aggregates and monomers indicate JC-1 accumulation within the mitochondria of healthy non-apopototic cells and distribution of JC-1 dye in the cytosol in mitochondrial membrane potential-compromised cells, respectively.

Fluorescence microscopy was also conducted in order to visualize the relative quantity of aggregate (red) fluorescence and monomer (green) fluorescence exhibited by the treated samples and the control samples.

### Ribonuclease A treatment

The experiments previously described whereby clonogenic survival and mitochondrial membrane potential were assessed following treatment with exosomes or UV-ICCM extracted from cells exposed to the UV bystander signal, were conducted in another permutation whereby exosomes, UV-ICCM, or UV-ICCM depleted of exosomes were treated with Ribonuclease A (RNase A) subsequent to UV-exposure and prior to the addition of the exosome fraction or UV-ICCM to clonogenic and/or mitochondrial membrane potential reporter cells.

Lyophilized RNase A was purchased from Sigma-Aldrich (Sigma-Aldrich, R6513) and reconstituted in sterile distilled water to a stock concentration of 10 mg/mL upon receipt. Working concentrations of 10 *μ*g/mL were diluted from the stocks and frozen at -20°C for future use. For RNase destined for incubation with pure UV-ICCM or with exosome fractions, the working concentration of RNase was added to a given volume of UV-ICCM or exosome fraction to produce a final concentration of 2 *μ*g/mL.

For ICCM, RNase was added to the UV-ICCM following 24 hour UV irradiation and after the UV-ICCM had been filtered through a 0.2 *μ*m pore filter. The UV-ICCM was incubated with RNase for 1 hour at 37°C prior to the addition of the ICCM-RNase solution into clonogenic reporter flasks. For exosome fractions, RNase was added to the exosome fraction following ultracentrifugation and resuspension in DPBS. The RNase was incubated with the exosome fraction for 1 hour at 37°C prior to the addition of the exosome-RNase solution into either flasks containing clonogenic reporter cells & 5 mL culture medium or into cell suspensions destined for mitochondrial membrane potential assessment.

### Western blot to validate exosome isolation

Western blots were conducted using protein extracted from both exosome fractions and from HCT116 p53+/+ whole cell lysate which had been exposed to UV photons emitted from non-irradiated or 0.5 Gy *β*-irradiated HCT116 p53 +/+ cells.

Proteins of interest included actin (42 kDa) and exosome-associated proteins, CD63 (non-glycosylated: 25 kDa, glycosylated: 30–70 kDa) and TSG101 (49 kDa). 10 *μ*g of protein was loaded into each well of a 10-well 12% bis-tris gel (Life Technologies) where the total volume in each well was 25 *μ*L. Proteins were transferred onto a 0.2 *μ*m nitrocellulose membrane (GE Health Sciences) and the membrane was blocked with 5% skim milk-TBST at room temperature for 60 minutes. The membrane was incubated with the primary antibody overnight at 4°C (anti-Actin rabbit polyclonal: Sigma-Aldrich A5060, 1:1000 in 5% milk-TBST; anti-CD63 rabbit polyclonal: Abcam ab68418, 1:1000 in 5% milk-TBST; anti-TSG101 mouse monoclonal: Abcam ab83, 1:1000 in 5% milk-TBST) followed by the secondary antibody for 60 minutes at room temperature (anti-rabbit, GE Amersham 45000679; anti-mouse, GE Amersham 45000682, 1:5000 in 5% milk-TBST). Following antibody incubation, blots were treated with enhanced chemiluminescence substrate (Thermo Scientific) prior to image acquisition (BioRad ChemiDoc MP, Image Lab 4.1 software). Protein band densities were quantified using image processing software, ImageJ. Protein from HepG2 and HeLa whole cell lysates were used as positive controls for CD63 and TSG101 protein expression, respectively. These whole cell lysates were chosen as positive controls since CD63 and TSG101 expression by HepG2 and HeLa cells had been validated by the manufacturer and thus their use as positive controls were recommended in the product data sheets supplied [36, 37].

### Transmission electron microscopy to validate exosome isolation

For visualization of samples using Transmission Electron Microscopy (TEM), exosomes were isolated in the same manner as described previously and subsequently resuspended in distilled H_2_O. Exosome suspensions were prepared on formvar-coated copper-palladium grids and negatively stained with uranyl acetate. Image acquisition was conducted using a JEOL 1200EX TEMSCAN electron microscope at the Health Sciences Centre Electron Microscopy Facility (McMaster University).

### Statistical analysis

Statistical differences among the clonogenic survival of cells subsequent to different treatments were assessed using a 1-way analysis of variance (ANOVA) test. Post-hoc analysis was conducted using Tukey’s honestly significant difference (HSD) test. A 1-way ANOVA was also employed to assess the statistical differences among the degree of mitochondrial membrane depolarization induced by various treatments. Tukey’s HSD test was employed for post-hoc analysis. Statistical analyses were conducted using GraphPad Prism 6 and SPSS Statistics 17.0.

## Results

### ICCM and exosomes from UV-exposed bystander cells

Reporter cells were subjected to cell conditioned medium or exosomes harvested from cells that were exposed to secondary UV biophotons to determine whether the UV signal emitted from *β*-irradiated cells could prompt a release of exosomes capable of eliciting a bystander response. It is emphasized that the term *UV-ICCM* throughout the text refers to culture medium conditioned by bystander cells which have been exposed to the *UV biophotons* emitted by *β*-irradiated cells.

#### Clonogenic survival following UV-ICCM transfer

**Fig 1:**
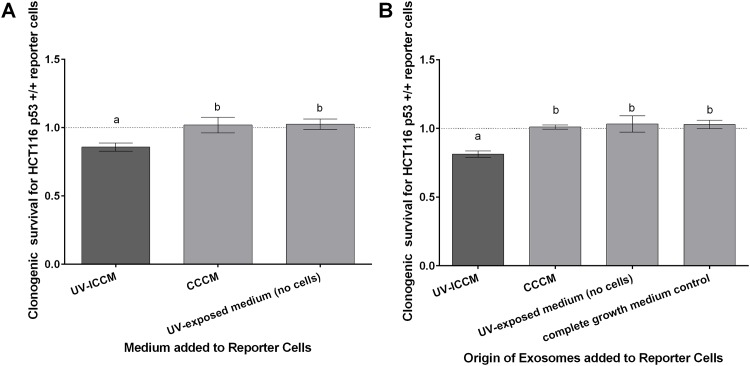
Reporter cells subjected to exosomes or conditioned culture medium from UV-exposed bystander cells (UV emitted from beta-irradiated cells). **(A)** Surviving fraction of HCT116 p53 +/+ cells cultured in UV-exposed ICCM or CCCM. Error bars represent SEM for 18 replicates (3 replicates for each of 6 independent experiments) for the UV-ICCM treatment and the CCCM control, and 9 replicates (3 replicates for 3 independent experiments) for the UV-exposed medium (no cell) control. **(B)** Surviving fraction of HCT116 p53 +/+ cells cultured in exosomes extracted from UV-exposed ICCM or CCCM. Error bars represent SEM for 18 replicates (3 replicates for each of 6 independent experiments) for the UV-ICCM exosome treatment and CCCM exosome control, and 9 replicates (3 replicates for 3 independent experiments) for the no cell control & medium only control. Letters (a,b,c) indicate significant differences between samples as assessed by means of 1-way ANOVA, 95% confidence level.

Upon transfer of ICCM from UV-exposed bystander cells to clonogenic-density reporter cells, a reduction in survival to 85.7% ± 3.0% was observed (Fig 1A). This reduction was significant when compared to the survival elicited subsequent to the transfer of medium from control cells not exposed to secondary UV biophotons and from cell-free cultures (UV-exposed medium only) to reporter cells (p<0.001).

#### Clonogenic survival following exosome transfer

The experiment was taken a step beyond that described in the previous section by extracting the exosomes from the UV-ICCM following irradiation and placing the exosome isolates, as opposed to the UV-ICCM, onto reporter cells. Fig 1B illustrates the ability of exosomes extracted from UV-ICCM to elicit a significant reduction in the clonogenic survival of HCT116 p53 +/+ reporter cells when compared to reporter cells which received control exosomes (p<0.012). The clonogenic survival of the UV-ICCM exosome-treated reporter cells was 81.2% ± 2.3%, whereas the survival of reporter cells receiving non-irradiated cell control exosomes, irradiated no-cell control exosomes, and medium only exosomes were 101.0% ± 1.5%, 103.2% ± 6.0% and 102.8% ± 3.1%, respectively.

When clonogenic survival of reporter cells receiving UV-ICCM and reporter cells receiving isolated exosomes are compared, it is found that the levels of cell killing induced by each of these treatments are comparable such that they are not significantly different (p = 0.493). This lack of difference in effect induced by UV-ICCM and exosomes may suggest that the cell killing effects observed as a result of UV-ICCM transfer are most likely due to the effect of exosomes since there does not appear to be an induced effect that is not accounted for by the exosomes.

#### Clonogenic survival following exosome-depleted CCM transfer

An additional experimental permutation was conducted whereby UV-ICCM and control CCM was ultracentrifuged to pellet and subsequently remove exosomes from the medium. The exosome-depleted UV-ICCM or CCCM was then placed onto reporter cells to determine the effect of exosome-free UV-ICCM and CCCM. The treatment of reporter cells with exosome-depleted UV-ICCM proved to induce significant (p<0.0001) cell killing in treated reporter cells to 80.1 ±3.0%. In contrast, the reporter cells treated with CCCM did not exhibit significant reductions in survival (100 ± 2.68%) when compared to the survival of reporter cells which received CCCM that was not depleted of exosomes (p = 0.78). We suggest that the effects observed here may be attributed the action of other soluble factors present in the UV-ICCM. During the UV exposure period (24 hour incubation where bystander cells were being exposed to UV), the exosomes released from the UV-exposed cells could very possibly act upon the same population of UV-exposed cells to prompt the release of cytokines, nitric oxides, and other soluble factors prior to isolation of exosomes from the UV-ICCM. This is suggested since the magnitudes of cell killing induced by UV-ICCM (with exosomes present) and that induced by exosomes extracted from UV-ICCM are comparable and thus do not support the idea that the exosomes and the other soluble factors present in UV-ICCM are acting in an additive manner, rather it is possible that one may lead to another. Despite these preliminary suggestions, further investigation will be required to properly interpret the implications of these results.

#### Mitochondrial membrane potential

**Fig 2:**
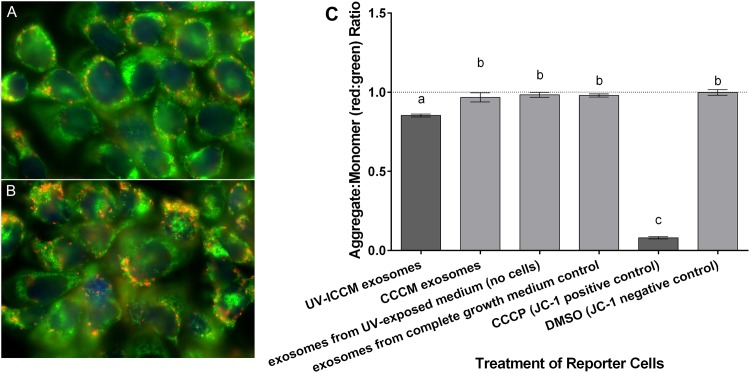
Fluorescence of JC-1 dye incubated with HCT116 p53 +/+ reporter cells which received. **(A)** exosomes extracted from ICCM treated with cell-emitted UV biophotons and **(B)** exosomes extracted from CCCM which did not receive UV biophoton irradiation. Fluorescence microscopy images were acquired using an Olympus IX81 microscope and Image Pro AMS 5.1 software. **(C)** Mitochondrial membrane potential observed in HCT116 p53+/+ cells following the receipt of exosome fractions extracted from UV-exposed bystander cells. The UV was emitted from directly-irradiated cells that were exposed to 0.5 Gy ^3^H *β*-radiation. Error bars represent SEM for a total of 18 replicates (6 replicates for each of 3 independent experiments). Fluorescence ratios were normalized to the DMSO negative control.

The effect of exosomes extracted from UV-ICCM upon the mitochondrial membrane potential of reporter cells was also assessed to determine the possibility for apoptosis induction in the exosome-treated reporter cells. Treatment of HCT116 p53 +/+ cells with exosomes extracted from UV-ICCM proved to induced a marked depolarization in the mitochondrial membrane of reporter cells (Aggregate to monomer ratio (AMR): 0.852 ± 0.009 (standard error of the mean)). The loss of mitochondrial membrane integrity in this population is illustrated in Fig 2A as a predominance of green monomer fluorescence. The appearance of red fluorescence in the UV-ICCM exosome-treated population was evidently diminished when compared to the red fluorescence demonstrated in the cell population treated with control exosomes (extracted from cells that were not exposed to UV) (Fig 2B). Fig 2C shows that the depolarization induced by the exosomes extracted from UV-ICCM was significantly different when compared to experimental controls and the assay negative control (DMSO) (p<0.0001). The membrane depolarization induced by the assay’s positive control (CCCP) (AMR: 0.080 ± 0.007) was significantly greater than that induced by the UV-ICCM exosomes (p<0.0001). However, the depolarization induced by exosomes extracted from UV-ICCM was still significant compared to negative controls, thus indicating that the treatment of reporter cells with exosomes may be able to induce apoptosis in a significant proportion of cells within the reporter population, albeit it is not able to generate as great of a response as other treatments such as CCCP.

### RNase-treated ICCM and exosomes from UV-exposed bystander cells

RNase was added into UV-ICCM or exosome fractions to confirm that the effects observed in response to reporter cell treatment with ultracentrifuged pellets were indeed induced by exosomes. More specifically, the intention was to determine the role of RNA-carrying exosomes in eliciting the responses observed.

#### Clonogenic survival following UV-ICCM transfer

**Fig 3:**
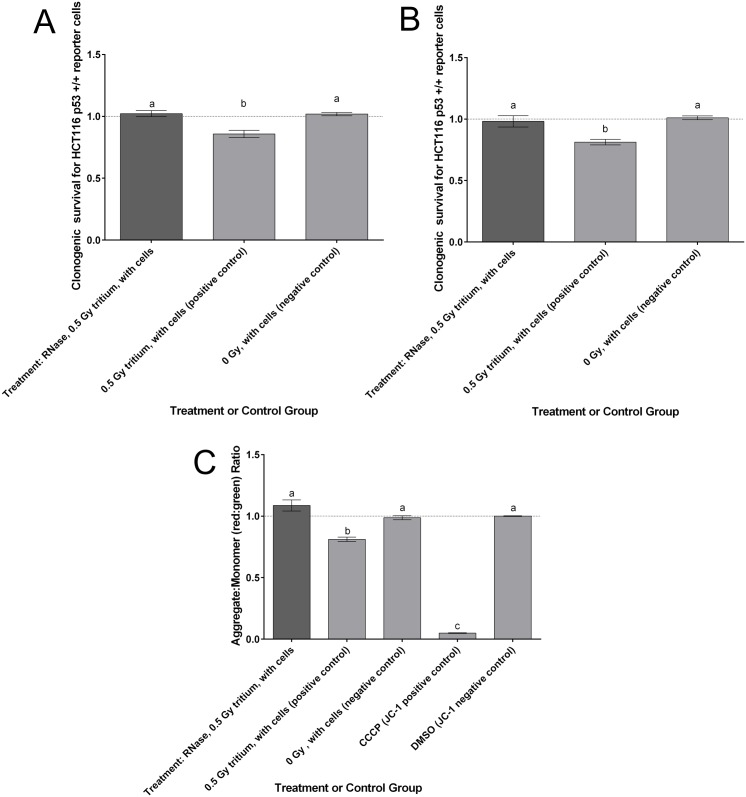
Reporter cells subjected to RNase-treated ICCM or exosomes derived from UV-exposed bystander cells. **(A)** Clonogenic survival of HCT116 p53 +/+ reporter cells receiving RNase-treated UV-ICCM, UV-ICCM, or CCCM. Error bars represent SEM for a total of 18 replicates (3 replicates for each of 6 independent experiments) for the 0.5 Gy positive control and 0 Gy negative control, and 9 replicates (3 replicates for 3 independent experiments) for the RNase-treated 0.5 Gy group. **(B)** Clonogenic survival of HCT116 p53 +/+ reporter cells following treatment of the reporter cells with RNase-treated UV-exposed exosomes, UV-exposed exosome fractions (no RNase treatment), or non-exposed exosome fractions. Error bars represent SEM for a total of 18 replicates (3 replicates for each of 6 independent experiments) for the 0.5 Gy positive control and 0 Gy negative control, and 9 replicates (3 replicates for 3 independent experiments) for the RNase-treated 0.5 Gy group. **(C)** Mitochondrial membrane potential (assessed via the incubation of cells with JC-1 mitochondrial potential dye) of HCT116 p53 +/+ reporter cells receiving RNase-treated or non-RNase-treated exosome isolates extracted from ICCM or CCCM. Error bars represent standard error of the mean for 6 replicates tested for each of three independent experiments (18 replicates total). Letters (a,b,c) represent significant differences between treatments as assessed by 1-way analysis of variance; post-hoc testing assessed using Tukey’s HSD test at the 95% confidence level.

Clonogenic survival was assessed in HCT116 p53 reporter +/+ cells that received UV-ICCM treated with RNase, UV-ICCM that was not treated with RNase, and CCCM which was not subjected to any exposure from secondarily-emitted UV. Fig 3A illustrates that RNase is effective in abolishing any negative cell killing effects that manifested in cells which received ICCM harvested from cells exposed to secondary UV radiation. Clonogenic survival in the RNase-treated population was not significantly different from that exhibited by the control cells receiving medium from non-UV-exposed cells (CCCM) (p = 0.972). In contrast, the receipt of UV-ICCM not treated with RNase was proven effective in reducing clonogenic survival significantly below the level of CCCM controls (p<0.0001).

#### Clonogenic survival following exosome transfer

Clonogenic survival of HCT116 p53 +/+ reporter cells was assessed following addition of exosome fractions to the reporter cell cultures. Exosome fractions were either extracted from UV-ICCM and subsequently treated with RNase, extracted from UV-ICCM and not treated with RNase (positive control), or extracted from conditioned medium of cells that were not exposed to UV biophotons (negative control). Fig 3B indicates the effectiveness of RNase in preventing a reduction in clonogenic cell survival for those exosome fractions which were extracted from UV-ICCM. The RNase treatment of the UV-ICCM exosome fraction was found to produce a statistically similar survival level to the level observed in the non-UV-exposed control (exosomes from CCCM) (p = 0.840). RNase treatment of the UV-ICCM exosomes proved to significantly assuage the proportion of cells killed when compared to the exosomes extracted from UV-ICCM that did not receive RNase treatment (p<0.0001).

In order to confirm that the effects observed in the reporter cells were indeed attributed to the action of RNase upon exosomes and not the direct action of the RNase upon the cells, controls were conducted whereby RNase was added into a non-irradiated cell population and a cell population which was directly-exposed with tritium. The results of these controls showed that RNase did not abolish nor assuage the cell killing observed in cells that were directly exposed to beta radiation (Cells exposed to 857.5 *μ*Ci ^3^H: 51.9 ± 7.7%, cells exposed to 857.5 *μ*Ci ^3^H + RNase: 45.4 ± 5.8%; p = 0.76). Furthermore, the addition of only RNase into cells resulted in a survival rate of 93.3 ± 9.7%. Thus, RNase treatment of these non-irradiated cells did not affect cell survival significantly when compared to the survival of non-irradiated cells that were not treated with RNase (p = 0.80).

#### Clonogenic survival following exosome-depleted CCM transfer

UV-ICCM, control CCM, and complete growth medium that was not conditioned with cells was ultracentrifuged to pellet and remove exosomes from the medium. Subsequently, the exosome-depleted UV-ICCM, CCCM and complete growth medium were treated with RNase prior to incubation with clonogenic reporter cells. The purpose of this control was to determine whether the RNA accounting for the observed bystander effects originated from the surface of the exosomes (contained within the medium) or from within the exosomes. After treating the clonogenic reporter cells with RNase-treated UV-ICCM depleted of exosomes, the survival observed was 84.6% ± 2.5%. Although the magnitude of cell death was slightly assuaged, the effect was not significantly different (p = 0.275) from that observed following the treatment of reporter cells with UV-ICCM depleted of exosomes that was not treated with RNase (80.1% ± 3.0%). While we cannot rule out a role for RNA present outside of the exosomes in eliciting part of the bystander response, the results suggest that the RNA-attributed effects observed in these experiments are predominantly driven by the RNA found within the exosomes. When reporter cells were exposed to RNase-treated CCCM depleted of exosomes and RNase-treated complete growth medium (not cell conditioned and depleted of exosomes), the resultant surviving fractions were 100.7% ± 3.2% and 100.1% ± 2.0%, respectively.

#### Mitochondrial membrane potential

Mitochondrial membrane potential in HCT116 p53 +/+ reporter cells was assessed following the treatment of reporter cells with RNase-treated exosome fractions or control exosome fractions. Thereafter, JC-1 dye was added to the reporters to identify the ratio of J-aggregates and monomers in reporter cells resultant to exosome treatment (Fig 3C). The treatment of reporter cells with RNase-treated exosomes isolated from UV-ICCM conferred a lack of significant change in the reporter cells’ mitochondrial membrane potential (AMR: 1.087 ± 0.045 (standard error of the mean)) when compared to the reporters which were treated with the assay’s negative control, DMSO (AMR: 1.00 ± 0.003) (p = 0.330) and when the RNase-treated group was compared to the non-irradiated control (AMR: 0.987 ± 0.016) (p = 0.163). In contrast, treatment of the reporter cells with exosomes isolated from UV-ICCM which were not treated with RNase proved to induce significant mitochondrial membrane depolarization (AMR: 0.812 ± 0.018) when compared to both of the negative controls (p<0.0001). The capability of RNase to abolish significant mitochondrial membrane depolarization, therefore, suggests that RNA, a factor which exosomes have been shown to carry [38], could be a factor that is responsible for eliciting a bystander response in reporter cells (those treated with exosomes extracted from UV-exposed cells).

### Validation of exosome isolation

To validate that the exosome extraction technique used in the current study was successful in isolating exosomes, western blots were conducted to identify exosome-associated proteins and transmission electron microscopy (TEM) was conducted to confirm the presence of and size of the microvesicles extracted by means of ultracentrifugation.

**Fig 4:**
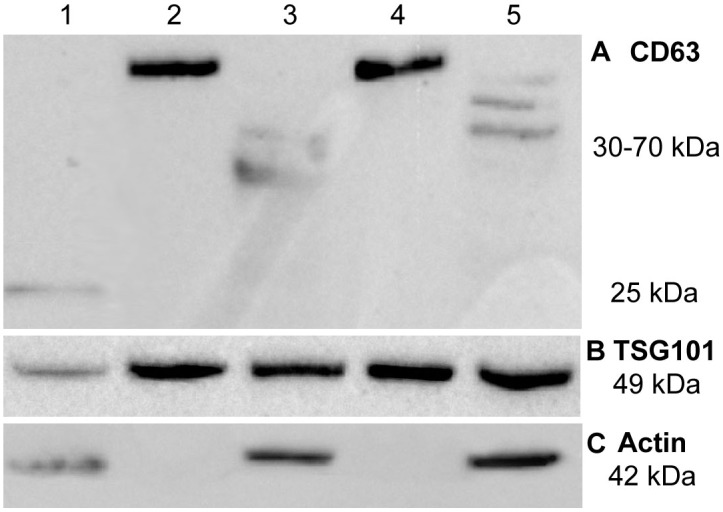
Protein bands acquired using western blots for expression of. **(A)** CD63 (glycosylated form: 30–70 kDa, non-glycosylated form: 25 kDa), **(B)** TSG101 (49 kDa), and **(C)** Actin (42 kDa). Lane 1: positive control (10 *μ*g protein from HepG2 whole cell lysate for CD63 and actin antibodies; 10 *μ*g protein from HeLa whole cell lysate for TSG101 antibody). Lane 2: exosomes extracted from HCT116 p53 +/+ CCCM. Lane 3: HCT116 p53 +/+ whole cell lysate not exposed to UV. Lane 4: exosomes extracted from HCT116 p53 +/+ UV-ICCM. Lane 5: HCT116 p53 +/+ whole cell lysate exposed to UV. All lanes contain 10 *μ*g protein each. The lack of actin expression demonstrated by lanes 2 and 4 indicate the absence of actin in exosome samples. Because actin is not required for exosome transport, the absence of actin in exosome samples is expected and indicates a lack of contamination by cellular debris in the exosome isolates.

Exosome-associated transmembrane protein, CD63, was expressed in its glycosylated form in exosome samples whereas whole cell lysates for the positive control (HepG2 cells) and for HCT116 p53+/+ cells expressed non-glycosylated and partially-glycosylated CD63, respectively (Fig 4A). The whole cell lysates extracted from UV-exposed cells appeared to undergo glycosylation to a greater extent than those which were extracted from non-UV-exposed cells. The observed expression of fully glycosylated CD63 was expected for exosome samples as found previously by Jelonek et al [39]. Furthermore, the absence of non-glycosylated or partially-glycosylated CD63 in the exosome isolates could be suggestive of a lack of contamination by cellular material in the exosome sample.

Upon assessing protein expression in exosome samples extracted from UV biophoton-exposed and non-exposed cells, it was shown that CD63 expression in exosomes from UV biophoton-exposed cells was significantly greater than that expressed in control exosomes (p = 0.028). The area under the curve, representative of band density as assessed by ImageJ, produced a normalized value of 9.8 ± 0.5 for exosomes extracted from UV biophoton-exposed cells and a value of 8.7 ± 0.1 for control exosomes (normalized to expression of HepG2 positive control). From these results, it can be suggested that the exposure of cells to UV biophotons may be responsible for initiating a release of exosomes from UV biophoton-exposed cells which is greater than the quantity of exosomes that would be secreted from non-UV-exposed cells. It is noted, however, that *whole cell lysates* subjected to secondary UV biophotons did not express CD63 to a degree that was significantly different from non-UV-exposed HCT116 cells (p = 0.779, normalized protein expression value: 1.64 ± 0.06 and 1.76 ± 0.29, respectively). Thus, the suggestion that UV biophoton exposure triggers the release of more exosomes requires further investigation before a sound conclusion can be drawn.

The second exosome-associated protein assessed in the current study, TSG101, is a cytosolic protein that is a component of the endosomal sorting complex required for transport and is involved in the generation of exosomes [40]. TSG101 expression was evident in both exosome samples and whole cell lysates for HCT116 p53 +/+ cells (Fig 4B). Upon comparing TSG101 expression between UV biophoton-exposed and non-exposed cells, the degree of protein expression did not differ among exosome isolates (p = 0.685, normalized protein expression: 3.9 ± 0.38 and 3.7 ± 0.44, respectively) nor among whole cell lysates (p = 0.182, normalized protein expression: 3.2 ± 0.25 and 2.6 ± 0.13, respectively). The lack of difference in TSG101 protein expression between exosome samples extracted from UV biophoton-exposed cells and control exosome samples casts doubt upon the idea that UV biophotons trigger the release of more exosomes from exposed cells. Rather, the difference in mitochondrial membrane potential and clonogenic survival induced by the exosomes from UV-ICCM may be explained, not by a greater quantity of exosomes, but a difference in the contents of the exosomes released by the UV-exposed cells and the control cells. This suggestion would of course require further investigation that is beyond the scope of this study at the present time.

Actin served as a negative control for protein expression in exosome samples such that its expression was not expected in exosome isolates but was expected in whole cell lysates [41, 42]. The results conferred in the current study agree with the aforementioned hypothesis since actin expression was present in HCT116 p53 +/+ whole cell lysates but not in exosome samples (Fig 4C).

**Fig 5:**
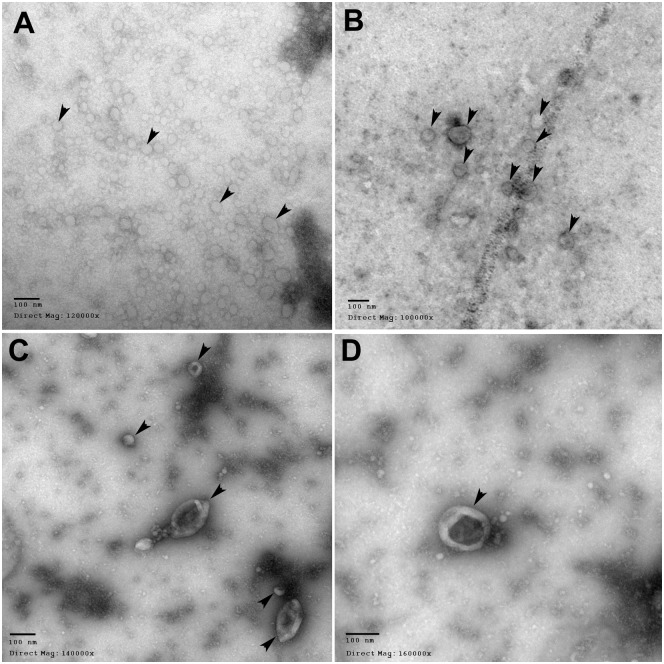
Transmission electron microscopy images illustrating the exosomes that were extracted from HCT116 p53 +/+ cells via ultracentrifugation. Exosomes are indicated by arrowheads. Scale bars in each of the four panels represent 100 nm. **(A)** Exosomes extracted from UV-exposed cells, direct magnification: 120 000x. **(B)** Exosomes extracted from non-UV-exposed control cells, direct magnification: 100 000x. **(C)** Exosomes from UV-exposed cells, direct magnification: 140 000x. **(D)** Exosome from UV-exposed cells, direct magnification: 160 000x.

Microvesicles possessing a diameter of approximately 100 *μ*m or less were readily visualized when TEM was employed to scrutinize the samples extracted via ultracentrifugation of ICCM and CCCM (Fig 5). The sizes of the visualized vesicles were within the range characteristic of exosomes (50–150 nm). From the observations made using TEM images and western blot analysis of exosome-associated proteins, it is possible to confirm with confidence that the exosomes isolation method used in the current study (ICCM and CCCM ultracentrifugation) was successful.

## Discussion

This study demonstrates novel evidence of a link between radiation-induced UV biophotons and exosomes (chosen for assessment to represent all soluble factors). To our knowledge, the current study is the first to suggest the reconciliation of these two bystander effect mediators. We previously demonstrated the modulation of the cell survival response in bystander cells exposed to UV biopohotons [13] whereby the directly-irradiated cell culture and the bystander culture were physically separated to the extent where there was no transfer of medium nor cell-cell contact at any point during the experiment. Even in the absence of medium transfer, co-culture, and direct cell-cell contact, the bystander effect was still elicited in the bystander cells receiving secondary UV signals. This observation thus introduced the idea that soluble factor release by directly-irradiated cells could not be the only mechanism driving the bystander response. Rather, there are either at least two separate bystander mechanisms (soluble molecules and biophotons), or soluble factors were being released by bystander cells subjected to UV signals emitted from directly-irradiated cells. In an effort to rationalize the observed bystander effects, we hypothesized the possibility of a link between UV biophotons and the release of exosomes from UV-exposed cells due to the literature that has recently emerged on the subject of exosome-mediated radiation bystander effects [17, 29, 32–34] and the demonstrated capacity for UV radiation to modulate exosome functions [28]. The observations made in the current study strongly support the existence of a bystander mechanism whereby the UV biophotons generated by directly-irradiated cells interact with bystander cells to induce the release of response-eliciting exosomes. That is not to say that soluble factors and exosomes are not released in response to direct cellular exposure to ionizing radiation. The results conferred in the current study simply illustrate that transfer of medium and direct cell-to-cell contact are not always required to elicit bystander responses in non-irradiated cells. We acknowledge that there are many soluble signalling factors that are involved in communicating the bystander effect and do not seek to invalidate those well-established mechanisms in any respect, we simply propose a plausible solution for situations in which bystander effects can be elicited in the absence of medium transfer and direct cell-cell contact. Alternatively, medium transfer and cell-cell contact are not required in another situation whereby volatile components from biological system can affect a nearby biological system [9, 10]. However, in the case of the current study, the two cell populations were physically separated such that even volatile species could not be shared or transferred.

*β*-irradiation of HCT116 p53 +/+ cells induced UV biophoton emission which was then subjected to bystander cells. The exosomes released from these UV-exposed bystander cells were subsequently isolated and used to assess downstream effects in reporter cells. These experiments show that the exosomes isolated from cells exposed to radiation-induced UV biophotons are capable of modulating the biological endpoints of cell death and mitochondrial membrane potential in reporter cells. Because exosomes are extensively diverse in regard to their content & abundance, and furthermore because their intravesicular contents can be modulated in response to various environmental conditions, it is difficult to establish the exact factors which are responsible for the effects that are observed in this particular case. It is suggested that a modulation of the RNA and protein cargo are likely to be influential in eliciting a bystander effect in cells receiving UV-exosomes compared to controls. However, we also did not rule out the idea that the UV biophotons could be responsible for initiating the release of exosomes from UV-exposed cells such that the samples extracted from UV-exposed ICCM would exhibit greater quantities of exosomes than the non-exposed controls.

Ribonucleic acid (RNA), particularly microRNA (miRNA), are carried within exosomes and have been proven to play a role in the radiation-induced bystander effect when x-rays have been used as the primary radiation source [17, 32]. Irradiation of a given cell can trigger the upregulation of specific miRNAs such as those involved in DNA damage response functions [32]. Through packaging of these miRNAs within vesicles such as exosomes, the miRNAs are easily exchanged intercelluarly and can subsequently elicit bystander effects in recipient cells. The current study shows that the induction of bystander effects in cells receiving exosomes extracted from UV-ICCM is quite possibly attributed to the action of the exosomes’ RNA contents. When the exosome pellet extracted from UV-ICCM were treated with RNase and subsequently incubated with reporter cells, reporter cells demonstrated a lack of stimulation represented by an absent cell death response and an abolished response in terms of mitochondrial membrane depolarization. These findings contrast significantly with those conferred following treatment of reporter cells with exosomes extracted from UV-ICCM that did not receive RNase treatment. Although we were not able to directly assess the permeability of exosomes to RNase, we conducted an experiment whereby exosome-depleted UV-ICCM was treated with RNase to determine whether the RNA accounting for the bystander effect originated from outside or within the exosomes. Our results suggest that RNA on the outside of exosomes may contribute to a portion of the effect. However, the effect appears to be attributed mainly to RNA contained within the exosomes.

It is possible that the mitchondrial membrane depolarization and cell death observed in reporter cells treated with exosomes extracted from UV-ICCM is the result of an upregulation of miRNAs detrimental to mitochondrial function or a downregulation of mito-protective miRNAs. To gain an accurate representation of the miRNAs that are involved in mediating the UV bystander effect, profiling of miRNA will be required in exosomes derived from both non-UV-exposed and UV-exposed media. It is possible, however, to confirm that the degradation of RNA was influential in abolishing significant bystander responses that are induced by the isolated exosome pellet under normal experimental conditions. This study has demonstrated the first evidence of RNA’s involvement in the UV-mediated bystander response.

This study used TEM to characterize the size of the vesicles isolated from ICCM by means of ultracentrifugation. TEM imaging was able to confirm that the size of the vesicles isolated in our experiments were characteristic of exosomes (50–150 nm). Furthermore, the expression of two exosome-associated proteins was assessed as per the guidelines recommended by Lotvall *et al*. [43]. Positive protein expression results were conferred for exosome associated proteins, CD63 and TSG101. Semi-quantitative assessment of CD63 protein expression suggested a possibility that UV biophoton exposure of reporter cells could trigger the release of more exosomes compared to non-exposed controls. This phenomenon of increased exosome abundance has also been observed by another research group using nanoparticle tracking analysis in glioma cell lines following exposure to x-radiation exposure [39]. However, the consistent expression of TSG101 protein across all exosome samples (both those isolated from UV-exposed cells and control cells) contrasts with the previous hypothesis and introduces the idea that cellular exposure to UV biophotons may induce a change in the contents carried by the exosomes as opposed to triggering the release of a greater quantity of exosomes. Although this suggestion has not yet been investigated in the current study which employs UV biophotons as the trigger, the published literature is supportive of the idea that radiation insult is capable of affecting the contents of excreted exosomes. Arscott and colleagues conducted molecular profiling of exosomes isolated from x-irradiated and non-irradiated U87MG cells to find that exosomes originating from x-irradiated cells exhibited 1308 and 209 mRNA changes 24 and 48 hours post-irradiation when compared to the mRNA sequences of non-irradiated cells [44]. Analysis of the protein contents of x-radiation-derived exosomes by Jelonek and colleagues revealed the presence of 236 proteins that were not detected in exosomes derived from non-irradiated FaDu cells. Among the proteins that were expressed in response to irradiation, the functions that predominated were those involved in cell division, transcription, and cell signalling [39]. The available literature which reports on exosomes derived from UV-exposed cells is limited. However, Cicero *et al*. investigated the exosome expression following direct UV-B irradiation of human keratinocytes and similarly concluded that the UV-B exposure did not affect the number of exosomes and rather hypothesized that ultraviolet radiation propagates its effects by altering the exosome composition [28]. Based upon obeservations made by previous investigators, it is not unreasonable to suggest that a stressor, such as secondarily-emitted UV biophotons, could initiate a change in the contents of exosomes released from UV-exposed cells. While this hypothesis has yet to be addressed, we consider the investigation of this inquiry an important future endeavour as it will provide valuable insight into the findings of the current work.

Although the work conducted in this study is restricted to *in vitro* investigations, it has generated results that have the potential to be expanded upon to elucidate the clinical relevance associated with exosomes isolated from UV-ICCM. The observation that exosomes extracted from UV-ICCM are capable of eliciting significant mitochondrial membrane depolarization can be considered an important first step in explaining a molecular mechanism for the radiation-related chronic fatigue and immune dysfunction syndrome (CFIDS). Mitochondrial membrane depolarization can be indicative of compromised ATP generation and subsequently manifest as the symptoms which characterize CFIDS [45]. The exertion of a systemic effect by exosomes, following even a targeted event such as an irradiation, makes plausible the suggested relationship between radiation exposure and CFIDS [46]. It will be crucial to explore this relationship further since the characterization and analysis of exosomes extracted from biological fluids may eventually be used as a predictor of many disease processes, including CFIDS.

## Limitations

A limitation of the current study involves the inconsistency in the variables between each of the endpoints investigated. The ratio of exosomes to reporter cells and the co-incubation times were different between the two assays used in the current study such that 9-day incubation with exosomes and 500 reporter cells were used in the clonogenic survival assay, while 1 hour exosome co-incubation and 2×10^6^ reporter cells were used in the mitochondrial membrane potential assay. Some of these differences were inevitable due to restrictions associated with assay-specific requirements and subsequently, these discrepancies between assay protocols result in the inability to conduct a valid and meaningful comparison of the magnitude of effect that the exosomes had upon each of the two endpoints assessed in the study. Despite the discrepancy, it is important to note that two widely different assays produced results that agree with each other when exposed to the same given treatment. This finding is important because we can be certain that the exosomes produced compatible effects, even under variable conditions.

## Conclusion

This paper was focused upon reconciling two apparently opposing bystander mechanisms. However, it was not meant to discount any other bystander mechanisms. These experiments show that exosomes capable of eliciting bystander effects are released from cells in response to exposure to non-ionizing UV signals emitted from directly-irradiated cells rather than being released as a direct result of the primary beta-irradiation itself. The exosomes extracted from UV-ICCM are effective in modulating clonogenic survival and mitochondrial membrane potential in bystander cells to a significant degree compared to exosomes extracted from CCCM harvested from non-UV-exposed cells. These effects could be abolished by the treatment of the exosome pellet with RNase. RNA is therefore considered influential in mediating the observed bystander effects. Similar expression of exosome-associated proteins among UV-ICCM-derived exosomes and control exosomes suggests that UV may not affect the quantity of exosomes released, rather it may elicit a modification of the cargo carried by the exosomes; it will be very important to investigate this hypothesis further. Most importantly, the study is the first to demonstrate a relationship between the radiation-induced bystander effect mediated by UV biophotons and exosomes. The significance of this result indicates that the transfer of medium is not always required for bystander signals to be communicated. Effect-eliciting soluble factors may still be generated in a bystander population which is subjected to the UV biophoton signals emitted from a directly-irradiated population.

## Supporting information

S1 File Raw Data File. This file contains the data corresponding to the results presented in the current manuscript.
(XLSX)
